# Development and cross-cultural testing of the International Depression Symptom Scale (IDSS): a measurement instrument designed to represent global presentations of depression

**DOI:** 10.1017/gmh.2017.16

**Published:** 2017-09-11

**Authors:** E. E. Haroz, J. Bass, C. Lee, S. S. Oo, K. Lin, B. Kohrt, L. Michalopolous, A. J. Nguyen, P. Bolton

**Affiliations:** 1 Department of Mental Health, Johns Hopkins Bloomberg School of Public Health, 624 N. Broadway, Baltimore, MD, USA; 2 Department of International Health, Johns Hopkins Bloomberg School of Public Health, 615 N. Wolfe St., Baltimore, MD, USA; 3 Aung Clinic, Yangon, Myanmar; 4 Thu Kha Nwe Specialist Clinic, Yangon, Myanmar; 5 Duke University, Duke Global Health Institute & Department of Psychiatry and Behavioral Sciences, Durham, NC, USA; 6 School of Social Work, Columbia University, 1255 Amsterdam Avenue, New York, NY, USA; 7 University of Virginia Curry School of Education, Charlottesville, VA, USA

**Keywords:** Assessment, depression, global mental health, validation

## Abstract

**Background:**

Self-report measurement instruments are commonly used to screen for mental health disorders in Low and Middle-Income Countries (LMIC). The Western origins of most depression instruments may constitute a bias when used globally. Western measures based on the DSM, do not fully capture the expression of depression globally. We developed a self-report scale design to address this limitation, the International Depression Symptom Scale-General version (IDSS-G), based on empirical evidence of the signs and symptoms of depression reported across cultures. This paper describes the rationale and process of its development and the results of an initial test among a non-Western population.

**Methods:**

We evaluated internal consistency reliability, test–retest reliability and inter-rater reliability of the IDSS-G in a sample *N* = 147 male and female attendees of primary health clinics in Yangon, Myanmar. For criterion validity, IDSS-G scores were compared with diagnosis by local psychiatrists using the Structured Clinical Interview for DSM (SCID). Construct validity was evaluated by investigating associations between the IDSS-G and the Patient Health Questionnaire (PHQ), impaired function, and suicidal ideation.

**Results:**

The IDSS-G showed high internal consistency reliability (*α* = 0.92), test–retest reliability (*r* = 0.87), and inter-rater reliability (*ICC* = 0.90). Strong correlations between the IDSS-G and PHQ-9, functioning, and suicidal ideation supported construct validity. Criterion validity was supported for use of the IDSS-G to identify people with a SCID diagnosed depressive disorder (major depression/dysthymia). The IDSS-G also demonstrated incremental validity by predicting functional impairment beyond that predicted by the PHQ-9. Results suggest that the IDSS-G accurately assesses depression in this population. Future testing in other populations will follow.

## Introduction

Task-sharing methods whereby non-specialists are trained to treat mental health disorders (Bolton *et al.* 2003; Patel *et al.* 2009), are viewed as a critical strategy to reduce the ‘treatment gap’ in global mental health. Treatment delivered by non-specialists has been shown to be effective for improving depression and other common mental health disorders (Bass *et al.* 2013; van Ginneken *et al.* 2013; Bolton *et al.* 2014).

A necessary first step in treating depression is identifying those individuals in need and who will likely benefit from treatment. As non-specialist workers do not have extensive training in recognizing the signs and symptoms of mental disorder, they typically rely on self-report instruments to conduct assessments. Many of these instruments were developed based on Western, and/or clinical populations (Hamilton, 1960; Beck *et al.* 1961; Zung *et al.* 1965; Radloff, 1977; Mulrow *et al.* 1995), and validity results have varied when using them in new settings and populations (Ali *et al.* 2016). Many of these instruments reflect DSM diagnostic criteria, a group of symptoms that appears to inadequately capture how depression is experienced around the world (Haroz *et al.* 2017). Some of these instruments have been successfully adapted (e.g. additional items, colloquial translations) and tested (Adewuya *et al*. 2006; Patel *et al.* 2008; Ghimire *et al.* 2013; Haroz *et al.* 2014). Other researchers have developed locally-specific screening instruments based on qualitative research in a particular context (Patel *et al.* 1997; Phan *et al*. 2004; Miller *et al.* 2006).

Both adapted and locally-specific instruments often perform well, but these approaches have limited generalizability (Ali *et al.* 2016). Moreover, instrument development and/or adaptation is a cumbersome process (Hollifield, 2002) that is not possible for many employers of non-specialist mental health care workers (e.g. non-governmental or community-based organizations) due to a lack of resources.

One option for addressing the limitations of existing instruments and processes of adaptation, is using an instrument that is less biased toward Western populations and more accurately reflects common ways of expressing depression around the world. An instrument that is based on empirical evidence of the commonalities in depression presentation globally should be more generalizable, and particularly useful for situations where local adaptation is not possible. We created such an instrument by empirically investigating symptoms associated with depression from a variety of populations around the world. The resulting instrument is the International Depression Symptom Scale that includes a General version (IDSS-G), which can be augmented with locally relevant symptoms (IDSS-L). In creating the IDSS we did not seek to create another depression instrument to diagnose DSM defined depression, but rather an instrument that better captures the experience of depression worldwide and reflects the constellation of symptoms associated with impaired functioning.

### Testing the IDSS

We tested the reliability, validity and clinical utility of the IDSS in a community sample of adults in Yangon, Myanmar. To evaluate whether the IDSS performed better than a commonly used standard screening instrument (translated but not adapted), we compared the IDSS with the Patient Health Questionnaire-9 (PHQ-9) on ability to predict impaired functioning (i.e. *incremental validity*). This testing is the first of a series of tests in different parts of the world with the goal of developing a reliable and valid instrument to measure depression that better reflects global commonalities in symptoms across and within different cultural contexts.

## Methods

### Development of the IDSS

The first step in the development of the IDSS involved a systematic review of qualitative research to identify common depression symptoms across geographic regions, gender, and contexts (Haroz *et al*. 2017). The second step involved a quantitative analysis using Item Response Theory (IRT) of the 15 symptom HSCL-25 depression scale administered in eight distinct cultural settings (Haroz *et al*. 2016). The new instrument combined symptoms that were common across multiple regions identified during the literature review with the best performing symptoms from the quantitative analysis. An expert panel of researchers and practitioners from the fields of global mental health, anthropology, psychiatric epidemiology, and psychiatry reviewed the draft instrument and additional revisions were made based on their feedback, which included adding symptoms from the DSM-5. These experts included two psychiatrists with extensive experience in global mental health, the former chair of the Department of Mental Health at Johns Hopkins Bloomberg School of Public Health who is a sociologist by training and has worked on developing measures of depression, two medical anthropologists who have been heavily involved in global mental health work for over 20 years, and a clinical psychology professor who has done extensive work with displaced populations.

The IDSS is a modular instrument with 29 items in the global measure (IDSS-G) and additional items added, based on qualitative research, when used in different settings (IDDS local; IDSS-L). The 29 items on the IDSS-G and the development process that supported each symptom's inclusion are provided in Table 1.

**Table 1.** Source of the supporting evidence for each symptom on the IDSS

Item	Qualitative review^a^	IRT analysis^b^	DSM-5
D01 sad	♦	♦	♦
D02 no interest	♦	♦	♦
D03 crying	♦	♦	
D04 hopeless	♦	♦	
D05 lonely	♦	♦	
D06 social withdrawal	♦		
D07 tired/fatigue	♦	♦	♦
D08 weigh too little	♦	♦	♦
D09 weigh too much	♦	♦	♦
D10 increased appetite	♦	♦	♦
D11 sleep problems	♦	♦	♦
D12 feeling trapped		♦	
D13 worry	♦	♦	
D14 worthless	♦	♦	
D15 headaches	♦		
D16 stomachaches	♦		
D17 general aches and pains	♦		
D18 anger	♦		
D19 thinking too much	♦		
D20 confused	♦		♦
D21 heart weakness	♦		
D22 palpitations	♦		
D23 heavy heart	♦		
D24 heart pressure	♦		
D25 heart pain	♦		
D26 psychomotor			♦
D27 concentration			♦
D28 imp function			♦
D29 suicide			♦

a Haroz *et al*. (2017).

b Haroz *et al.* (2016).

#### Study procedures

All participants completed the assessment battery and were evaluated by a local psychiatrist. To assess test–retest and inter-rater reliability, *n* = 54 randomly selected participants were interviewed a second time. The same interviewer who administered the initial interview (*n* = 24) or a different interviewer (*n* = 30) conducted the re-interview.

##### Psychiatric evaluation

Local psychiatrists conducted evaluations for each participant within 2–5 days of the initial assessment. Diagnoses from these interviews were treated as the primary criterion for validity analysis. The first *n* = 40 study participants were interviewed by psychiatrists in pairs (with independent ratings) in order to establish inter-rater reliability. The remainder of participants were interviewed by psychiatrists working individually.

#### Participants

Study participants were recruited from two medical clinics in Yangon, Myanmar. We purposively sampled participants from these clinics as there were reported high rates of psychiatric disorders (~30–40%), indicating a high likelihood we would include both participants with mental disorders, and some without. To be included in the study, participants had to be a clinic patient and over the age of 18. Exclusion criteria consisted of active psychosis or the presence of a major developmental delay. All participants provided informed verbal consent. The study was approved by the Johns Hopkins Internal Review Board (IRB #6011) and the Ethics Review Committee of the Department of Medical Research (Lower Myanmar).

#### Measures

*The IDSS-G* is a 29-item self-report measure. Participants were asked to indicate how often in the last 2 weeks they had experienced each symptom in the measure. Responses options ranged from 0 ‘none of the time’ to 3 ‘almost all the time.’

PHQ-9 (Kroenke *et al*. 2001) is a nine-item self-report measure that asks participants how often in the past 2 weeks the symptom bothered him/her. Response options ranged from 0 ‘not at all’ to 3 ‘nearly every day.’ The PHQ-9 is a commonly used measure of depression and has been found to be valid in a variety of low-resource settings (Lotrakul *et al*. 2008; Marc *et al*. 2014; Zhong *et al*. 2014), although it had not been previously tested in Myanmar.

*Structured Diagnostic Interview for DSM-IV* (SCID; Spitzer *et al*. 1995) is a semi-structured interview designed for use by trained mental health professionals to facilitate diagnosing DSM Axis I disorders (American Psychiatric Association, 2000). For the current study only major depressive disorder (MDD), dysthymia and generalized anxiety disorder (GAD) were evaluated. Diagnosis of GAD was included due to its high co-morbidity with depression (Almeida *et al*. 2012) and overlap in diagnostic criteria (American Psychiatric Association, 2013).

A *Local measure of functional impairment* was previously developed and validated among Burmese refugees displaced in Thailand (Haroz *et al.* 2014). The measure includes tasks that men (16 items) and women (23 items) do to care for themselves, their families and their communities. Participants were asked how much difficulty he/she had in the last 4 weeks compared with other men/women of similar age. Response options ranged from 0 ‘no difficulty’ to 4 ‘often cannot do.’

Functional impairment in this study was used as a validity criterion for two reasons. First, it is often the major domain that is adversely affected among people suffering from mental health symptoms and provides a way to assess the presence of symptoms and their impact on people's daily lives. Second, assessing the association of symptoms with impaired functioning broadened our criteria beyond DSM diagnosis.

#### Translation

The assessment instruments were translated and back-translated by the local study coordinator and a local psychiatrist. Review of all translations took place as part of training the interviewers and psychiatrists. Each item was reviewed during each of the trainings. When minor problems with wording or phrasing arose, we discussed as a group, and settled on wording by consensus. No major problems with translations were identified during the trainings. In addition, a subset of participants (*n* = 30 men and *n* = 30 women) was asked to complete a cognitive interview to assess face validity and the comprehension of select items from the IDSS-G. For each symptom question, participants were asked: (1) *Please describe the meaning of this question in your own words*; (2) *Is there any part of this question you don't understand or that does not make sense?*; (3) *Can you tell me what thought you had when deciding your answer choice*?; and (4) *Was this question easy or difficult to answer*? Sixteen items on the IDSS-G had been previously tested in a similar population (see Haroz *et al*. 2014) and were not included in the cognitive interview. The remaining 13 items from the IDSS-G were part of the cognitive interviews.

#### Interviewers and psychiatrists

Eight local interviewers administered the full assessment using mobile devices and facilitated the cognitive interviewing. Interviewers were people from the community with previous experience doing data collection. Interviewers were trained in study procedures, research ethics, and a safety protocol, during a 3-day training prior to data collection. The interviewers administered the IDSS verbally using tablets to record participants’ responses. This was done as literacy rates were inconsistent and we wanted a uniform interview procedure for all participants involved in the study.

Four local psychiatrists conducted the clinical interviews using the SCID. All psychiatrists had medical degrees obtained from medical schools in Myanmar. Three had been practicing for more than 5 years, while the fourth was finishing residency. All psychiatrists attended a 3-day SCID training prior to data collection. Each psychiatrist was given a handout with the DSM-IV criteria for the three study disorders (MDD, dysthymia, and/or GAD). They were instructed to use the SCID to inform their clinical judgment as to diagnosis.

#### Analysis

Average summary scores for the IDSS-G, PHQ-9 and functional impairment measures were generated. For the IDSS-G, two items were not included in summary scores: ‘difficulty doing your usual activities at home or work’ and ‘thoughts of wanting to kill yourself.’ These items were included in the instrument to assess severity and safety risk. All analyses were done using STATA-13 (StataCorp, 2013) and Mplus 7.3 (Muthén & Muthén, 1998–2012).

### Reliability

We examined: (1) Exploratory Factor Analysis (EFA) with geomin rotation, (2) internal consistency reliability, and (3) test–retest and inter-rater reliability for the IDSS-G. The EFA examined factor loadings and item uniqueness. Cronbach's alpha (*α*) (Cronbach, 1951) was used for internal consistency reliability. Pearson's correlation coefficients (*r*) were calculated for test–retest reliability. Correlations of |0.7| or above are considered very strong, |0.4| to |0.69| strong, |0.3| to |0.39| moderate, |0.2| to |0.29| weak, and less than |0.2| are considered negligible (Cohen, 1988). Inter-rater reliability was assessed using intra-class correlation (ICC) by comparing scores from the first interview to scores on re-interview (done by a different interviewer). ICCs >0.75 are considered excellent; 0.40–0.75 fair to good; and <0.40 poor (Fleiss, 1986).

To establish the reliability of psychiatrist diagnosis, inter-rater reliability between pairs was calculated using a Kappa statistic. A Kappa of <0 indicates less than chance agreement; 0.01–0.20 slight agreement; 0.21–0.40 fair agreement; 0.41–0.60 moderate agreement; 0.61–0.80 substantial agreement; and 0.81–0.99 indicates almost perfect agreement (Viera & Garrett, 2005).

### Validity

We examined construct, criterion, and incremental validity. Construct validity is defined as the degree to which a scale measures the theoretical construct that it was designed to measure and is correlated to other related constructs. Criterion validity is defined as the association of a scale to a criterion variable (i.e. psychiatric diagnosis and functional impairment) (Allen & Yen, 2002). Incremental validity refers to the ability of a measure to increase predictive ability beyond another measure (Sackett & Lievens, 2008).

For construct validity, we use Pearson's correlation coefficients (*r*) and Spearman's correlation coefficients (*ρ*), to assess the strength of relationships between the IDSS-G and age, gender, functioning, PHQ-9, and the single functional impairment and suicidal ideation items. Based on evidence in the literature, we hypothesized that higher scores on the IDSS-G would be associated with increasing age (Jorm, 2000; Kessler *et al.* 2003; Bromet *et al.* 2011); female gender (Nolen-Hoeksema *et al*. 1999; Bromet *et al.* 2011); greater impairments in functioning (Ormel *et al.* 2008; Kessler & Bromet, 2013), and suicidal ideation (Nock *et al.* 2008). As both the IDSS-G and the PHQ-9 measure depression, we expected that scores on these measures would be highly correlated. Evidence for these associations would support construct validity.

For criterion validity, scores on the IDSS-G of participants diagnosed with a SCID disorder were compared with those without a disorder. This was followed by comparing any depressive disorder to no disorder. Criterion validity would be supported if IDSS-G scores were substantially and significantly higher among participants with any diagnosis and/or a depression disorder (depression/dysthymia) compared to those without a disorder. Determination of whether the difference of means between diagnostic categories was statistically significant was done using logistic regression.

### Incremental validity

Incremental validity was assessed using a series of linear regression models in which variables were added stepwise to predict functional impairment. Model 1 examined the impact of age. In model 2, suicidal ideation was also added, followed by inclusion of the PHQ-9 in model 3, and addition of the IDSS-G in model 4. Incremental validity would be supported if scores on the IDSS-G significantly predicted functional impairment (*p* *<* 0.05), above and beyond the impact of age, suicidal ideation and scores on the PHQ-9, as measured by a statistically significant increase (*F* test) in the *R*^2^ statistic when comparing model 4 with model 3 (Sackett & Lievens, 2008). We also examined the degree of collinearity between scores on the IDSS-G and PHQ-9 using a variance inflation factor (VIF). VIFs of 5 or greater are usually cause for concern (Craney & Surles, 2002) and indicate that variables are highly collinear.

### Clinical utility

Receiver operating curves (ROC) were used to compare the area under the curve (AUC), for the IDSS-G and PHQ-9 across diagnostic comparisons. ROC curves plot the true positive rate (sensitivity) against the false positive rate (1-specificity). An AUC of 0.5 (50% sensitivity and 50% specificity) indicates that the test is of no diagnostic utility, while an AUC of 1.0 (100% sensitivity and 100% specificity) indicates perfect prediction of the criterion. AUC values of 0.50–0.70 indicate low accuracy; 0.70–0.90 moderate accuracy, and above 0.90 high accuracy (Fischer *et al*. 2003). An optimal cut-off point was generated for the IDSS-G based on maximizing sensitivity and specificity (Liu, 2012).

## Results

### Descriptive statistics

Overall *N* = 151 people were interviewed using the IDSS-G and associated measures; *n* = 2 refused to participate in the SCID evaluation and *n* = 2 had data that were mistakenly erased during uploading; leaving a final analytic sample of *n* = 147. Two-thirds of the participants were women (*n* = 95; 63.8%) and ages ranged from 18 to 81 with a mean age of 47.5.

Average scores on the IDSS-G ranged from 0 to 2.44 with a mean of 0.72 (s.d. = 0.49). Scores on all of the measures were positively skewed, indicating that most participants reported few symptoms and good functioning (Table 2). The positive skew across the sample was most likely a result of our sampling method, which aimed to have both participants who were well-functioning (i.e. no disorder), and participants who were less well.

**Table 2.** Mean scores and frequencies for scales on assessment battery and frequency of SCID diagnoses

Measure	*N*	*M*	Range	s.d.	Skew
IDSS-G	147	0.72	0–2.44	0.49	1.07
PHQ-9	146	0.67	0–3	0.63	1.46
Functioning	147	0.61	0–2.43	0.60	1.08
SCID diagnosis (*N* = 147)			*N* (%)		
Any disorder			71 (48.3)		
Depression			31 (21.1)		
Dysthymia			39 (26.5)		
GAD			22 (15.0)		
None of these disorders			63 (42.9)		
Co-morbidity (two or more)			24 (16.3)		

Based on psychiatrist diagnosis with the SCID, *n* = 31 people met criteria for MDD, *n* = 39 people for dysthymia, and *n* = 22 for GAD (Table 2). Of those with a comorbidity (*n* = 24), *n* = 18 had MDD and dysthymia; *n* = 4 had MDD and GAD; and *n* = 2 with dysthymia and GAD. One participant was diagnosed with all three disorders (Table 2). A little less than half of the total sample (*n* = 63; 42.9%) had none of the evaluated SCID disorders.

### Cognitive interviewing

Most questions were easily understood, with the exception of ‘feeling weakness in your heart’ and ‘feeling as though your heart was heavy’ (*n* = 15 and 7 found it difficult to understand, respectively). Most of the items were believed to be related to mental health problems with the exception of the items representing somatic complaints. For example, the majority of people talked about ‘stomach pain’ being related to medical problems or eating spicy food. Only one person mentioned that stomach pain could come from stress. The item ‘other bodily aches and pains’ also overwhelmingly was reported to be related to physical and medical issues, with most respondents describing having this symptom after being sick or having a medical issue (*n* = 29), working too much (*n* = 15), or being caused by cold weather (*n* = 14). The meanings of the items ‘feeling weakness in your heart,’ ‘heart palpitations’ ‘feeling pressure on your heart’ and ‘pain in your heart’ were described as related to medical problems as well.

### Reliability results

#### Factor analysis

We explored one- to five-factor solutions. The three-factor solution was selected as the most appropriate model based on loadings and what made theoretical sense. The majority of items (Table 3) loaded on the first factor, and include symptoms related to depressed mood, social isolation, and cognitive impairment. The items related to appetite and weight loaded on the second factor. The third factor included many of the somatic symptoms such as ‘headaches’ and all of the heart-related items. Four items do not appear to load on any of the factors and these include: ‘tired/fatigue,’ ‘problems with sleep’ and ‘stomach aches,’ and ‘other aches and pains.’

**Table 3.** Factor loadings for items on the IDSS-G

	F1	F2	F3
D01 sad	0.713*	0.107	0.091
D02 no interest	0.688*	−0.001	−0.034
D03 crying	0.579*	0.287*	0.058
D04 hopeless	0.565*	−0.059	0.248*
D05 lonely	0.748*	0.006	−0.062
D06 social withdrawal	0.745*	0.096	−0.077
D07 tired/fatigue	0.282	0.325*	0.359*
D08 weigh too little	0.351	0.731*	−0.013
D09 weigh too much	0.041	−0.594*	0.167
D10 increased appetite	0.070	0.609*	0.275*
D11 sleep problems	0.276*	0.242*	0.278*
D12 trapped	0.903*	−0.019	−0.063
D13 worry	0.692*	−0.055	0.015
D14 worthless	0.565*	−0.001	0.165
D15 headaches	−0.023	0.238*	0.578*
D16 stomach_aches	−0.254	0.298*	0.344*
D17 other_aches	0.198	0.205	0.209
D18 anger	0.549*	−0.199*	0.109
D19 thinking too much	0.784*	−0.197	0.012
D20 confused	0.843*	−0.042	−0.063
D21 heart_weakness	0.067	0.154	0.543*
D22 palpitations	0.079	0.257*	0.600*
D23 heavy_heart	0.009	−0.059	0.910*
D24 heart_pressure	0.112	−0.014	0.861*
D25 heart_pain	−0.033	0.340*	0.550*
D26 psychomotor	0.608*	0.261*	−0.135
D27 concentration	0.605*	0.060	−0.086
D28 imp function	0.598*	0.084	0.144
D29 suicide	0.674*	0.345*	0.050

*p *<* 0.05.

#### Internal consistency reliability and item analysis

Cronbach's alpha was high for the IDSS-G (*α* = 0.92). Analysis of item-level correlations supported dropping only one item, ‘weighing too much,’ as the item was negatively correlated with all other items. Alpha for the PHQ-9 was lower (*α* = 0.83) and item analysis did not support the removal of any items.

#### Test–retest reliability

Re-interviews by the same interviewer were performed within 2–11 days of the initial administration of the IDSS-G (mean = 3.8 days; s.d. = 2.17). Visual inspection of the graph depicting the relationship between IDSS-G scores at the first interview and re-interviews indicated that a linear relationship fit the data well. The correlation between average scores on the first interview with average scores on the re-interview was *r* = 0.87, indicating a strong positive relationship and good test–retest reliability. The PHQ-9 also showed good test–retest reliability (*r* = 0.88).

#### Inter-rater reliability

On average, re-interviews with different interviewers were done 10.2 days (s.d. = 5.3; *range:* 2–19 days) after the initial administration of the IDSS-G. The average ICC across interviewers for the IDSS-G was *ICC* = 0.90 with a 95% CI of (0.79–0.95), indicating high inter-rater reliability. Inter-rater reliability was lower for the PHQ-9 (*ICC* = 0.77; 95% CI 0.53–0.89). Kappas between each pair of psychiatrists indicated substantial to almost perfect agreement for all diagnosis (range: *κ* = 0.64 for no diagnosis *v*. any diagnosis to *κ* = 1.00 for GAD *v*. no diagnosis), with the exception of the dysthymia rating in pair 1 for which only fair agreement was achieved (*κ* = 0.38).

### Validity

#### Construct validity

Table 4 displays the polychoric correlation matrix for: (1) IDSS-G; (2) age; (3) gender; (3) functional impairment measure; (4) PHQ-9; (5) functional impairment item; and (6) suicidal ideation item. Construct validity was supported by a very strong correlation between the IDSS-G and the PHQ-9 (*r* = 0.78) and strong correlations between the IDSS-G and functional impairment scale (*r* = 0.56), and item (*ρ* = 0.65), and suicidal ideation item (*ρ* = 0.65).

**Table 4.** Correlations of IDSS-G and other measured variables

	IDSS-G	Age	Gender	Functioning measure	PHQ-9	Function item	Suicide item
IDSS-G	1.00						
Age	−0.16	1.00					
Gender	0.17	−0.06	1.00				
Functioning measure	0.56*	−0.17*	−0.11	1.00			
PHQ-9	0.78*	−0.18*	0.06	0.50*	1.00		
Functioning item	0.65*	−0.16	−0.05	0.48*	0.62*	1.00	
Suicide item	0.65*	−0.40*	0.09	0.50*	0.56*	0.56*	1.00

*p *<* 0.05.

#### Criterion validity

**Fig. 1.**
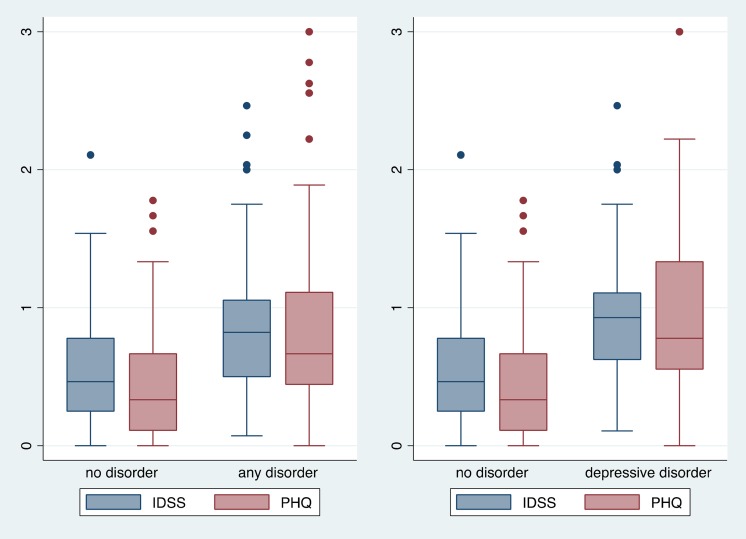
Box plots of scores on the IDSS and PHQ-9 over SCID diagnoses.

Average scores on the IDSS-G were higher among all disorder classifications (any disorder: mean = 0.87, s.d. = 0.47; depressive disorder: mean = 0.93, s.d. = 0.49; GAD: mean = 0.73, s.d. = 0.40) compared with participants classified as not having any of the SCID disorders (mean = 0.55, s.d. = 0.43). Logistic regressions indicated statistically significant differences between the mean score on the IDSS-G for participants classified as having any disorder and MDD/dysthymia compared with participants with none of these disorders. Results were similar for the PHQ-9 with higher average scores across disorder classifications compared with those classified as having no disorder (Fig. 1).

#### Incremental validity

Table 5 presents results from the incremental validity investigation. The final model (model 4) included all variables from model 3, as well as average scores on the IDSS-G. Thirty-four percent of the total variance in impaired functioning was explained by the variables in model 4 (additional 7% explained in model 4 compared with model 3). Results from model 4 indicated that after controlling for age, suicidal ideation and scores on the PHQ-9, every unit increase on the IDSS-G was associated with a 0.47 increase in impaired functioning. In model 4, both the IDSS and the PHQ-9 had VIFs of 2.8 and tolerances of 0.39. Moreover, after adding the IDSS-G, the PHQ-9 was no longer significantly associated with impaired functioning. The *F* test comparing models 3 and 4 indicated a statistically significant increase in *R*^2^ when the IDSS-G was added to the model (*p* = 0.001), thus supporting incremental validity of the IDSS-G. Reversing the order the variables were added (i.e. adding the IDSS-G to model 3, then the PHQ-9 to model 4), resulted in no change in the variance explained between models 3 and 4, and the IDSS-G remained significant.

**Table 5.** Effects of measured variables on impaired functioning presented as beta coefficients

Model	*β* (s.e.)	*t*
Model 1		
Age	−0.008 (0.01)	−2.21*
Model 2		
Age	−0.004 (0.01)	−1.22
Suicidal ideation^a^	0.71 (0.15)	4.75**
Model 3		
Age	−0.003 (0.01)	−0.92
Suicidal ideation	0.25 (0.16)	1.56
PHQ-9	0.37 (0.08)	4.78**
Model 4		
Age	−0.003 (0.01)	−0.92
Suicidal ideation	0.22 (0.16)	1.40
PHQ-9	0.12 (0.11)	1.21
IDSS-G	0.47 (0.14)	3.35**

a For the purposes of the incremental validity testing, the item related to suicide ideation was dichotomized meaning that 0 = none of the time and 1 = some, most and almost all of the time.

*p *<* 0.05.

**p *<* 0.001.

##### Sensitivity analysis

We performed a sensitivity analysis to check how stable our coefficients were in models 3 and 4 when using smaller samples. If collinearity is a problem with the IDSS and PHQ-9 score variables, we would expect increases in s.e. of the estimate, making it hard to reject the null hypothesis that there is no difference between the scores on each scale. Reducing our sample size would amplify this result. To test this, we randomly split our sample into equal groups of *n* *=* 102 each. We re-ran models 1–4 in these subsamples. In model 3 of the first sample, average scores on the PHQ-9 were significant (*b* *=* 0.32; *p* = 0.014, with an *R*^2^ = 0.30). In model 4, the PHQ-9 became not significant and the IDSS was significant (*b* *=* 0.48; *p* = 0.022, with an *R*^2^ = 0.35) with a 5% increase in *R*^2^. In the second subsample, we found similar results: model 3 indicated scores on the PHQ-9 were significant (*b* *=* 0.34; *p* = 0.007, with an *R*^2^ = 0.26) and model 4 indicated that scores on the PHQ-9 were not significant and the IDSS was significant with a 5% increase in *R*^2^ (*b* *=* 0.44; *p* = 0.027, with an *R*^2^ = 0.31). Despite smaller sample sizes, parameter estimates and changes in *R*^2^ remained consistent with our overall findings suggesting scores on the PHQ-9 and IDSS are not problematically collinear.

#### Clinical utility

**Fig. 2.**
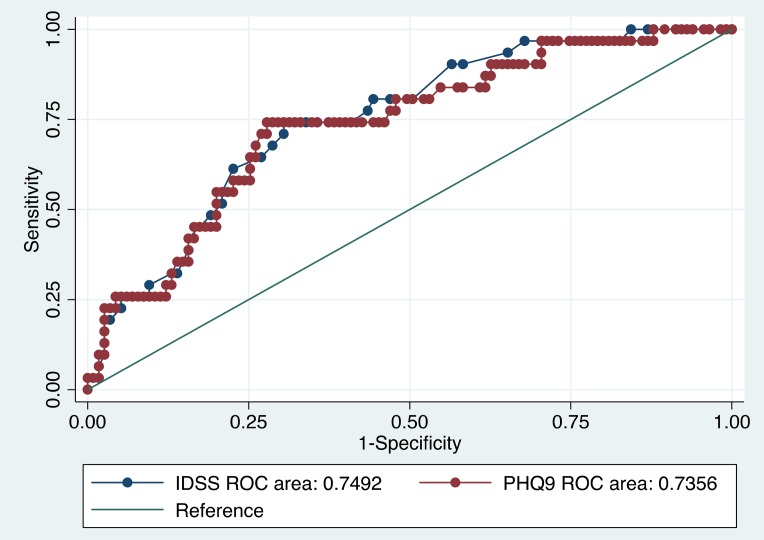
ROCs for IDSS and PHQ-9 using a diagnosis of depressive disorder.

The IDSS-G had an AUC of 0.72 (95% CI 0.63–0.81) for the comparison on of any disorder to no disorder and an AUC of 0.75 (95% CI 0.66–0.83) when comparing depressive disorders (MDD/dysthymia) to no disorder (Fig. 2). The AUC results for the PHQ-9 were similar indicating moderate accuracy for both scales, across diagnostic comparisons. The PHQ-9 had an AUC of 0.74 (95% CI 0.65–0.82) for the comparison of any disorder to no disorder; and an AUC of 0.74 (95% CI 0.64–0.83) for the comparison of a depressive disorder (MDD/dysthymia) to no disorder (Fig. 2). We identified an optimal cut-off score for the IDSS-G of 0.56, which corresponded to a sensitivity and specificity of 73% and 67% for any *v*. no disorder, and 77% and 67% for a depressive disorder *v*. no disorder.

## Discussion

The present study examined the reliability, validity, and clinical utility of the newly created IDSS-G, a self-report instrument developed based on an empirical investigation into the signs and symptoms of depression that occur in populations around the world. Reliability statistics for the IDSS-G were either equivalent or slightly higher than those of the commonly used PHQ-9. High correlation between the two self-report measures, as well as high correlation between the IDSS-G and both functional impairment and suicidal ideation, support the instrument's construct validity. Criterion validity was demonstrated by significantly higher IDSS-G scores among individuals assessed by a psychiatrist as having a disorder than among those assessed as having no disorder. Both the IDSS-G and PHQ-9 showed low to moderate diagnostic utility for detecting DSM defined disorders; however, the IDSS-G predicted functional impairment slightly better than the PHQ-9 in this setting.

These results suggest that both the IDSS-G and PHQ-9 are both suitable screening instruments to detect DSM-defined depressive disorders in this context, particularly by non-specialist providers who lack the training to conduct extensive diagnostic interviews. Given that both the SCID and the PHQ-9 are derived from the DSM, it is not surprising that the PHQ-9 would perform well against SCID diagnoses. The IDSS-G, on the other hand, was developed based on cross-cultural descriptions of depression that capture symptoms beyond those included in the DSM. The ability of the IDSS-G to perform similarly well against SCID diagnoses is an important minimal standard supporting its use. However, that the IDSS-G slightly, but significantly, *outperformed* the PHQ-9 at predicting functional impairment – a major outcome of interest in mental health – is a key study finding demonstrating the important contribution of this new instrument over existing instruments. However, given the wide scale use of the PHQ-9 in studies around the world, this finding would need to be replicated in other settings and populations.

While, the IDSS-G slightly, but significantly, predicted higher levels of functional impairment compared with the PHQ-9 in this sample, suggesting that the Western DSM model of depression, as reflected by the PHQ-9, may be inadequate in this population. It may be that the IDSS-G includes elements of disorder that are even more closely related to functioning than the DSM diagnostic criteria or core symptoms of Western depression. This finding would need to be replicated in other settings to justify the use of the IDSS over a shorter and comparable instrument like the PHQ-9.

Local adaptation of existing instruments is typically a critical element of instrument testing in new settings for just this reason – existing models of depression are specific to Western presentations, and instruments based on these models, are likely to miss relevant local expressions of distress. Our findings suggest that some of these missing ‘local’ symptoms are, in fact, symptoms relevant to depression across multiple cultures that are simply not reflected in the DSM (Haroz *et al*. 2017). In this case, using an instrument developed based on global presentations of depression appears to be more likely to capture locally relevant impairment than a Western measure that reflects DSM diagnostic criteria such as the PHQ-9.

Because the IDSS-G was developed to be a global instrument, we did not conduct preliminary adaptation before testing it a new context. Despite studies showing that adapted Western-based instruments can be reliable and valid in other contexts (Bass *et al.* 2008; Haroz *et al.* 2014; Rasmussen *et al*. 2014; Ali *et al.* 2016), very few studies have actually evaluated the impact of adaptation on scale validity. Jayawickreme *et al*. (2012) conducted a study looking at the incremental validity of Western instruments that incorporated local idioms of distress. In this study, instruments that incorporated local idioms predicted functional impairment above and beyond simple translations of well-established Western measures. The authors stress the importance of doing brief ethnographic work to inform scale adaptation (Applied Mental Health Research Group, 2013).

The development of the IDSS-G does not diminish the importance of locally relevant signs and symptoms of distress. When identified, these local indicators are important to include, as they represent common ways of expressing distress in each setting (Keys *et al.* 2012; Kohrt *et al.* 2014), may more saliently communicate illness, be less stigmatizing, and useful for measuring treatment success (Kohrt *et al.* 2014). The incremental validity of the un-adapted IDSS-G over the PHQ-9 suggests that it may be a better measure of depression in non-Western contexts, both as a starting point for local adaptation and when preliminary qualitative work and adaptation is not feasible.

### Limitations

The study was conducted in a single site in Myanmar and involved a non-random sample in an urban setting, many of whom had medical illness. It is possible that the sampling strategy explains why we did not identify gender and age differences that would be expected based on the literature (Nolen-Hoeksema *et al*. 1999; Van de Velde *et al*. 2010; Ferrari *et al.* 2013). Likewise, the overrepresentation of participants with a medical illness may help to explain why the somatic items performed differently, even though these items are common globally (Haroz *et al*. 2017). Many of the symptoms included in the IDSS-G are based on English translations of depression symptoms found in qualitative research. However, despite efforts to find accurate translations of symptoms, direct translation often results in overlapping terms that do not necessarily fully capture the original meaning (Nichter, 2010). It is possible that some symptoms on the translated IDSS-G may not fully capture how distress is conveyed locally, pointing to the need for local adaptation when possible. Finally, it remains unclear as to whether the IDSS-G is diagnostically superior to a locally developed measure of depression that incorporates idioms of distress.

### Conclusion

Overall, the findings show that the IDSS-G is a reliable and valid depression instrument in Yangon, Myanmar. Incremental validity found the IDSS-G to be a better able to predict impaired functioning than the PHQ-9. We speculate that this may be true for other non-Western populations. Further development and testing of the IDSS-G in multiple populations is necessary to determine whether this cross-culturally derived instrument is preferable to current standard instruments developed in the West.
